# Transcriptome Analysis of “*Candidatus* Liberibacter solanacearum” in Its Psyllid Vector, *Bactericera cockerelli*

**DOI:** 10.1371/journal.pone.0100955

**Published:** 2014-07-03

**Authors:** Freddy Ibanez, Julien Levy, Cecilia Tamborindeguy

**Affiliations:** 1 Department of Entomology, Texas A&M University, College Station, Texas, United States of America; 2 Department of Horticultural Sciences, Texas A&M University, College Station, Texas, United States of America; Natural Resources Canada, Canada

## Abstract

“*Candidatus* Liberibacter solanacearum” (Lso) is an emergent pathogen of carrots in Europe and solanaceous plants in North and Central America and New Zealand. This bacterium is closely related to other pathogenic *Candidatus* Liberibacter spp., all vectored by psyllids. In order to understand the molecular interaction of this pathogen and its psyllid vector, *Bactericera cockerelli*, Illumina sequencing of psyllid harboring Lso was performed to determine if this approach could be used to assess the bacterial transcriptome in this association. Prior to sequencing, psyllid RNA was purified and insect and bacterial rRNA were removed. Mapping of reads to Lso genome revealed that over 92% of the bacterial genes were expressed in the vector, and that the COG categories Translation and Post-translational modification, protein turnover, chaperone functions were the most expressed functional categories. Expression levels of selected Lso genes were confirmed by RT-qPCR. The transcriptomic analysis also helped correct Lso genome annotation by identifying the expression of genes that were not predicted in the genome sequencing effort.

## Introduction

“*Candidatus* (*Ca.*) Liberibacter solanacearum” (Lso) is a Gram-negative α-proteobacterium plant pathogen vectored by the potato psyllid *Bactericera cockerelli* Sulc (Hemiptera: Triozidae) [1] and the carrot psyllids *Trioza apicalis* Förster (Hemiptera: Triozidae) [2] and *Bactericera trigonica* Hodkinson (Hemiptera: Triozidae) [3]. This and closely related bacteria, such as “*Ca*. Liberibacter asiaticus” (Las), are emerging as major plant pathogens, all of them vectored by psyllids [4], [5], [6], [7], [8].

“*Candidatus* Liberibacter solanacearum” is the causative agent of potato zebra chip in the USA, Mexico, Guatemala, Honduras and New Zealand [4], [5], [9], [10], [11]. This bacterium thrives in two very different environments, plant phloem and insect vectors. The *in silico* annotation of the 1.26 Mbp Lso genome yielded 1,192 putative proteins, as well as 3 complete rRNA operons (16S, 23S and 5S) and 45 genes encoding tRNAs [12]. However, nothing is known about the genetic program expressed in either host. Similarly, approximately 35% of the putative proteins encode hypothetical proteins [12] and their expression and function remain unknown. Recently, different Lso haplotypes were identified [13], [14], [15], [16] but only the genome of Lso haplotype B is currently available.

Insects in the order Hemiptera are major vectors of plant pathogens. Many vectors are phloem feeding insects that depend on obligate endosymbionts to complement their diet with essential amino acids [17]. The dependency of the insect and obligate endosymbiont often led to host-symbiont co-speciation [18], [19], [20] and genome reduction of the obligate endocellular symbiont [21], [22]. Often, these insects also associate with facultative symbionts that might confer other benefits such as protection against natural enemies [23] or abiotic stresses [24], and nutritional benefits [25]. Therefore, insects can harbor a variety of microorganisms that might interact and affect each other as well as their insect host.

The study of Lso, other vector-borne plant pathogenic bacteria, and insect endosymbionts is often limited by our inability to culture those microorganisms. Previously, global studies were mainly based on candidate genes identified by *in silico* predictions [26], [27]. However, this approach has several limitations such as problems predicting small genes or correctly identifying UTRs. Next-generation sequencing is revolutionizing our ability to monitor global gene expression from key organisms [28]. The possibility of obtaining millions of reads at low cost opens the door to the study of complex systems in which the RNA from the target organisms cannot be purified. For instance, RNA from *B. cockerelli* harboring Lso would contain a majority of insect RNA as well as RNA from the associated bacteria, *Carsonella ruddii* (psyllid obligatory endosymbiont), Lso and any other associated symbiont [29]. For each of these organisms over 95% of the RNA would be rRNA and tRNA, the rest corresponds to mRNA. Finally, prokaryotic mRNA lacks the poly(A) tail found in mature eukaryotic mRNA and cannot be purified, which has been a major hurdle for global gene expression of bacteria.

The aims of this study were three-fold. Firstly, we conducted the first global gene expression study of a *Liberibacter* species in its vector. To our knowledge this is the first next-generation sequencing study of a vector-borne plant pathogenic bacterium in either host. Secondly, we aimed at compiling a list of genes potentially involved in vector-pathogen interaction that might be used as candidate genes to disrupt transmission by the insect. Finally, our study was aimed at helping the annotation effort of this pathogen by acquiring supporting evidence of the expression of predicted genes, by improving the prediction of the bacterial genes by helping identify UTRs, and by identifying other putative genes not found by the prediction software.

## Materials and Methods

### Insects

*Bactericera cockerelli* colony carrying Lso haplotype B was maintained on tomato plants in 14″×14″×24″ insect cages (BioQuip, Rancho Dominguez, CA, USA) at room temperature and photoperiod of 12∶12 h (L:D).

### Identification of endosymbionts

Endosymbionts associated with *B. cockerelli* used in this study were identified as described in [29]. Briefly, DNA from 18 insects was extracted following the protocol of [30], [31] and presence of Lso, *C. ruddii*, *Wolbachia* and S-endosymbiont was tested by PCR [29]. Identification of Lso haplotype was performed using the Lso-SSR-1F and Lso-SSR-1R primers [14].

### RNA purification and sequencing

Total RNA from 32 mg of adult *B. cockerelli* was extracted using RNeasy Mini kit (Qiagen, Valencia, CA) following the manufacturer's instructions. Genomic DNA contamination was eliminated by DNase treatment with RNase-Free DNase (Qiagen, Valencia, CA). The total RNA quantity and purity was determined using a Biophotometer plus (Eppendorf, Hamburg, Germany) and RNA integrity was visualized by electrophoresis in agarose gels at 1.2% stained with ethidium bromide.

To remove insect large ribosomal RNA (rRNA) from total RNA, probes against *B. cockerelli* 18S rRNA and 28S rRNA [32] were designed (Table 1) and synthetized (Integrated DNA Technologies). Probes were resuspended at 100 µM and denatured prior to their use by heating 5 min at 65 C and placed immediately on ice. Six µg of total RNA were subjected to ribosomal RNA depletion using RiboMinus Transcriptome Isolation kit (Life Technologies, Carlsbad, CA) combined with 100 pmol of each psyllid specific probes and 100 pmol of bacterial rRNA specific probes from the RiboMinus Transcriptome Isolation kit following the manufacturer's instructions with the subsequent modifications: incubation of total RNA and probes was performed for 30 min at 37°C, the hybridization buffer was diluted to half and two rounds of Dynabead recovery were performed. Removal of rRNA was verified by bioanalyzer (Agilent Technologies).

**Table 1:** Probes used for ribosomal RNA 18S and 28S depletion from *B. cockerelli* RNA sample.

Name	Sequence
Probe1BC28S	5-Biotin-TCGAGTAAGTAAGGAAACGAT-3
Probe2BC28S	5-Biotin-TGGAGTCAAGCTCAACAGGGT-3
Probe1BC18S	5-Biotin-ATTCAGTTATTCTATGCACACA-3
Probe2BC18S	5-Biotin-ACTAAGTCATCGGAGGAACTT-3

Insect mRNA was recovered using Dynabeads mRNA Purification kit (Life Technologies, Carlsbad, CA). Since Lso genome has 35.24% G+C content [12], bacterial mRNA could also be recovered in this sample.

PolyA purified RNA and the rest of the RNA (rRNA depleted and polyA depleted, later called depleted RNA) from one pool of psyllids were submitted to the Texas AgriLife Genomics and Bioinformatics Core Facility in Texas A&M University for sequencing. The library from the polyA purified RNA was prepared using the RNA-TruSeq Library kit (Illumina) and the library from the depleted RNA was prepared using ScriptSeq RNA kit (Epicenter Biotechnologies, Illumina, Madison, WI). Both single end libraries were combined in a 60∶40 ratio (depleted RNA:polyA purified RNA) and sequenced using a half lane of 100 base-length read chemistry on Illumina Hi-SEQ system. Sequencing data have been deposited in NCBI GEO's database (accession number: GSE57808).

### Bioinformatic data analysis

The Illumina pipeline programs for sequence processing was used to produce the fastq files, sort libraries and remove the barcodes and adaptors. CLC genomic workbench 4.8 platform was used for read mapping and RNA-seq analysis.

Reads were mapped to Lso haplotype B (PRJNA61245) [12], *C. ruddii* (PRJNA172734, primary endosymbiont of psyllids, genome sequenced from *Heteropsylla cubana*) [33], and *Wolbachia pipientis* endosymbiont of *Drosophila melanogaster* (NC_002978) [34] genomes. Mappings to the bacterial genomes were performed using the CLC Genomic Workbench Map Reads to Reference tool with the following parameters: mismatch cost 2, insertion cost 3, deletion cost 3, length fraction 0.5, and similarity 0.8.

The transcriptional abundance of each gene was determined using the RPKM (reads per kilobase of transcript per million mapped reads) value. RPKM was computed by CLC Genomics Workbench 4.8 platform using Lso genome as a reference with the following mapping settings: minimum length fraction 0.9, minimum similarity fraction 0.8, maximum number of hits for a read 10, and type of organism prokaryote.

### Expression analyses by RT-qPCR

To validate bioinformatics analyses, three cDNA synthesis reactions were performed from the same rRNA depleted RNA sample used for Illumina sequencing. For each cDNA synthesis reaction, 500 ng of RNA were processed as template using Verso cDNA Synthesis kit (Thermo, Waltham, MA) with random hexamers primers following the manufacturer's instructions. The RT-qPCR reactions were performed using SensiFAST SYBR Hi-ROX Kit (Bioline, Taunton, MA) according to manufacturer's instructions. Each reaction contained 5 ng of cDNA, 250 nM of each primer and 1X of SYBR Green Master Mix, the volume was adjusted with nuclease-free water to 10 µL. The real-time PCR program was 95°C for 2 min followed by 40 cycles at 95°C for 5 sec and 60°C for 30 sec. Primers were designed using Primer3 web [35] (Table 2). Real-time PCR assays were performed using an Applied Biosystems ABI 7300 real-time PCR Thermocycler (Applied Biosystems) according to manufacturer's recommendations. For RT-qPCR, two technical replicates for each of the three synthetized cDNAs were performed, with negative controls in each run. The threshold cycles (Ct) values and the efficiency of each primer set for RT-qPCR were determined using LinRegPCR software [36]. The relative expression of each gene [2^-(CTtarget gene-CTnormalizer gene)^] was estimated by normalizing transcript levels of genes of interest to the internal control gene (Lso *recA*) expression values.

**Table 2:** Primers used in gene expression analysis.

Annotation	Sequence	Primer efficiency (%)	Amplicon size (bp)
Transcriptional regulator CarD family protein	CKC_01945 F	96.4	103
	5-GGGACTTATACCGCACAGAAA-3		
	CKC_01945 R		
	5-TTTACAGCCGCAACTTCTCT-3		
C4-dicarboxylate transporter DctA	CKC_02250 F	96.9	103
	5-TCTCTCTCTCTCTCGTTGGTAAA-3		
	CKC_02250 R		
	5-GCAGCTCGCATAACGATAGA-3		
Polynucleotide phosphorylase	CKC_01280 F	96.5	116
	5-CGGACATATGCTGTTGGTAAGA-3		
	CKC_01280 R		
	5-GGAAACAACGGCCGAATAGA-3		
Alpha-ketoglutarate decarboxylase	CKC_03205 F	96.7	101
	5-CAGGGAGTTCAGCAAGGTATAG-3		
	CKC_03205 R		
	5-ATAGAACGAGGCGGTTTATTCA-3		
ABC transporter nucleotide binding/ATPase protein (iron)	CKC_05615 F	96.3	105
	5-TGCACTTAAAGGCGATATGATCT-3		
	CKC_05615 R		
	5-TGCCTATAACGACCCATCATTAC-3		
Flagellar motor switch protein G fliG	CKC_00530 F	96.5	103
	5-GAAGTCGATCCTACGGCTATTG-3		
	CKC_00530 R		
	5-GAACACTTGCTCCAATTGATGG-3		
Hypothetical protein	CKC_03925 F	95.9	110
	5-CGTATTACGCCAGAAGATACCC-3		
	CKC_03925 R		
	5-GCATTAGGACCAGGAGATGAAA-3		
Hypothetical protein	CKC_03935 F	95.8	103
	5-AGAAGAGACGCTAGCAGAGA-3		
	CKC_03935 R		
	5-GACCGAAGAATGTCCCACTAC-3		
Type I secretion system ATPase, PrtD	CKC_02260 F	97.1	126
	5-AGCAAAGGAACGCCACCAAT-3		
	CKC_02260 R		
	5-ACACTGGGATCAAAGGCGTT-3		
Pilus assembly protein	CKC_00705 F	95.1	103
	5-TGAATGCACTGGAACGAGCA-3		
	CKC_00705 R		
	5-AAACGCGTTCCACCTGATCT-3		
Recombinase A *recA*	CKC_05085 F	96.9	103
	5-AGGCAAACTTTCACCCATATCGCC-3		
	CKC_05085 R		
	5-ACAGATATGCTGGTGCGTTCAGGA-3		

To assess the expression of the same genes in different biological samples, three new pools of ten adult psyllids harboring Lso haplotype B were collected. For each pool, total RNA was purified using Trizol (Life Technologies, Carlsbad, CA) following manufacturer's instructions. RNA was treated with Turbo RNase-free DNase (Life Technologies, Carlsbad, CA). Five hundred ng of each total RNA sample were used as template to synthetize cDNA as previously described. Reverse transcription qPCR reactions were performed as previously described. For each tested Lso gene, two technical replicates were performed, with negative controls in each run.

Gene expression data from bioinformatics analyses and RT-qPCRs were compared using Pearson's correlation coefficient. Relative expression for each target gene using *recA* as normalizer was obtained; for bioinformatic gene expression the formula: RPKM _target gene_/RPKM _recA_ was used, and for RT-qPCR 2^-(CTtarget gene-CTnormalizer gene)^ formula was used.

### Verification of putative newly annotated genes

Primers for candidate genes were designed using Primer3 web tool (Table 3) [35] to amplify the putative full length gene. For each candidate, a PCR was performed using DNA obtained from psyllids as previously described. Reactions were performed using PrimeSTAR Max DNA Polymerase (Clontech, Mountain View, CA) according to the manufacturer's recommendations with the following conditions: 94°C for 2 min; followed by 30 cycles of 94°C for 30 sec, 60°C for 30 sec, and 72°C for 1 to 3 min (1 min per amplified kb); and a final extension at 72°C for 3 min. PCR products were examined by gel electrophoresis, purified using PureLink Quick Gel Extraction kit (Life Technologies, Carlsbad, CA) and cloned into the pGEM-T vector using the pGEM-T Easy cloning kit (Promega, Madison, WI) following the manufacturer's recommendations. Plasmid DNA from three clones were purified using PureLink Quick Plasmid Miniprep Kit (Life Technologies, Carlsbad, CA) and sequenced by Eton bioscience Inc. Obtained sequences were compared to the Lso genome sequence and the consensus prediction. Expression of the candidate gene was checked by RT-PCR as previously described (primer sequences used for RT-PCR are reported in Table 3).

**Table 3:** Primers used to validate sequence and expression of putatively new annotated genes.

Name	Sequence	Amplicon size (bp)
**Primers used to verify candidate gene DNA sequence**		
CKC_SecB-like F1	5-ACCTGTTTCTATTTCCATAGGCA-3	779
CKC_SecB-like R1	5-TCAAATGATGGTTGGAGGATCA-3	
CKC_Hyp protein F1	5-TCCATATAAGCAACAATCATTCCC-3	600
CKC_Hyp proteinR1	5-TTACTTAATGACTGAGGAGAGGAA-3	
CKC_PGDH-like F1	5-AAAGCAAAGAAACAGCATCCTC-3	1219
CKC_PGDH-like R1	5-AGAAGAGATGGCTCGCATTATAG-3	
**Primers use to test cDNA expression by RT-PCR**		
CKC_SecB-like F2	5-AACCCGCAATACAAATCAATG-3	178
CKC_SecB-like R2	5-TGCGAAATGTGTTCTTTTGG-3	
CKC_Hyp protein F2	5-CGAGCGAACTTTCAACCTTT-3	108
CKC_Hyp proteinR2	5-TCTGCGTAATAAGCTTGTTGGA-3	
CKC_PGDH-like F2	5-AGCCAGGTGTTCGCATTATC-3	206
CKC_PGDH-like R2	5-CTTTTCTTGGGCTTCCACTG-3	

## Results and Discussion

### Bacteria associated with *B. cockerelli* used in this study

As expected, PCR analysis confirmed the presence of *C. ruddii*, the primary endosymbiont of psyllids (Figure S1A). Similarly, presence of *Wolbachia* and Lso were detected (Figure S1B). Analysis of Lso haplotype showed that only haplotype B was present in the insects used in this study (Figure S1C). The S-endosymbiont was not detected (data not shown). These results were in accordance with previous periodical monitoring of endosymbionts and Lso haplotype associated with this colony.

### Purifying and sequencing RNA from bacteria associated with *B. cockerelli*

**Figure 1:**
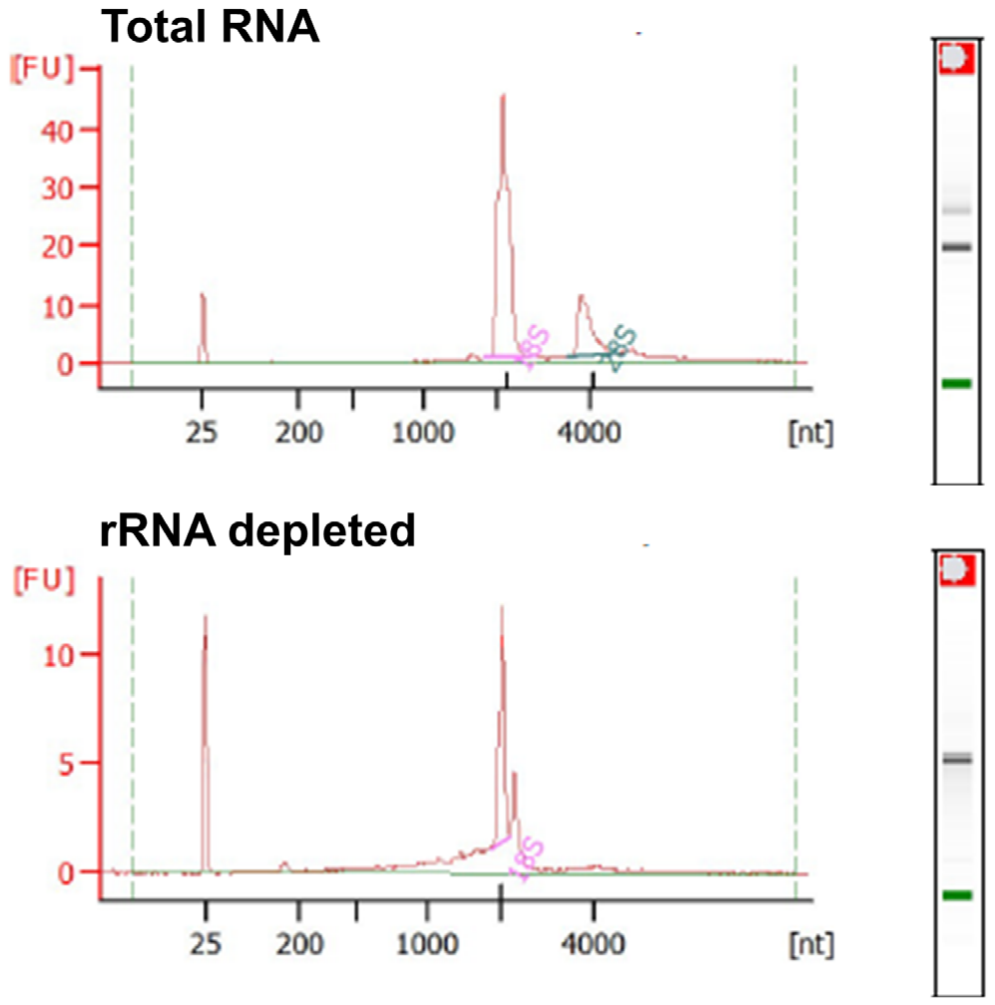
Bioanalyzer assay of total RNA and depleted RNA used in this study. After rRNA removal the peak associated with 28S rRNA disappeared and the area of the peak associated with 18S rRNA went from 85.4 to 9.

To analyze Lso transcriptome in its vector, RNA from one pool of psyllids harboring Lso was purified. Removal of insect rRNA was performed using *B. cockerelli* specific probes designed using the sequences previously published [32]. After rRNA removal, we observed a significant reduction of the 28S rRNA and 18S rRNA (Figure 1). According to the bioanalyzer results, the 18S rRNA and the 28S rRNA represented 73.1% of the total area in the total RNA sample, whereas they represented 24% of the total area in the RNA depleted sample.

Over 70 million reads were obtained (27,816,627 reads from the polyA purified RNA sample and 43,053,357 from the depleted RNA sample) (Table 4), which corresponded to the 40∶60 ratio on which both libraries were combined for sequencing. Reads were mapped to the genomes of Lso haplotype B [12], *C. ruddii* primary endosymbiont of the psyllid *H. cubana* [33], and *W. pipientis* from *D. melanogaster* [34]. *Carsonella ruddii* from *H. cubana* was chosen for the analysis for multiple reasons. Firstly, it was found that all analyzed *C. ruddii* genomes showed perfect conserved synteny [33]. Secondly, *H. cubana* is not gall forming species, therefore it should have diet requirements more similar to *B. cockerelli* than gall-forming psyllids. Remarkably, the majority of the reads mapping bacteria mapped to Lso (46.4%) (Table 5). It is probable that once the genome of *C. ruddii* and *Wolbachia* associated with *B. cockerelli* are available a higher number of reads could be matched to these bacteria.

**Table 4:** Summary statistics of the global sequencing and read mapping.

	PolyA purified RNA	Depleted RNA	Total
Total reads	27,816,627 (39.3%)	43,053,357 (60.7%)	70,869,984
Mapped to bacterial genomes	353,466 (46.6%)	405,642 (53.4%)	759,108

**Table 5:** Detailed read mapping to bacterial genomes.

	Genome size (bp)	Total mapped reads (% of reads mapped to bacterial genomes)	Mapped reads from polyA purified RNA library	Mapped reads from depleted RNA library
Lso genome	1,258,278 bp	352,751 reads (46.4% of mapped reads)	120,449 reads (34.2%)	232,302 reads (65.2%)
*C. ruddii* genome	166,163 bp	242,643 reads (32% of mapped reads)	145,243 reads (59.9%)	97,400 reads (40.1%)
*Wolbachia* genome	1,267,782 bp	163,714 reads (21.6% of mapped reads)	57,774 reads (35.3%)	105,940 reads (64.7%)

A total of 352,751 reads mapped to Lso haplotype B genome (120,449 reads from the polyA purified RNA sample and 232,302 reads from the depleted RNA sample) (Table 5). It is interesting to note that 1/3 of the mapped reads were from the polyA purified RNA library as Lso genome has 35.24% G+C content (Lin et al. 2011). On the other hand, a total of 242,643 reads mapped *C. ruddii* genome (GC content ∼15%) and 60% of them were from the polyA purified RNA library. *Carsonella ruddii* is one of the bacterial endosymbionts with the strongest AT bias. For example, the aphid primary endosymbiont genome, *Buchnera aphidicola* has around 30% G+C content [37]. It is accepted that because of this strong AT bias, a large portion of *B. aphidicola* mRNA can be purified using polyA purification methods. Recently, the transcriptome of *B. aphidicola* associated with the soybean aphid *Aphis glycines* was analyzed by RNA-seq from polyA purified RNA [38]. In that study, the authors measured expression of more than half of the *Buchnera* genes. Aphids used in that study also harbored Wolbachia, but very few transcripts from Wolbachia genes were identified. However, in a study of *Wolbachia* transcriptome in nematodes [39] more than 95% of the bacterial genes were found expressed when RNA was not polyA purified. Similarly, in our analysis of *B. cockerelli* transcriptome [32], less than 45,000 reads of over 75 million reads mapped *C. ruddii* genome. Therefore, it appears that even for bacteria with an extreme AT bias such as *C. ruddii*, the use of RNA depleted samples or a combination of polyA purified and RNA depleted samples should be considered when studying their transcriptomes.

### Analysis of Lso transcriptome in its vector

**Figure 2:**
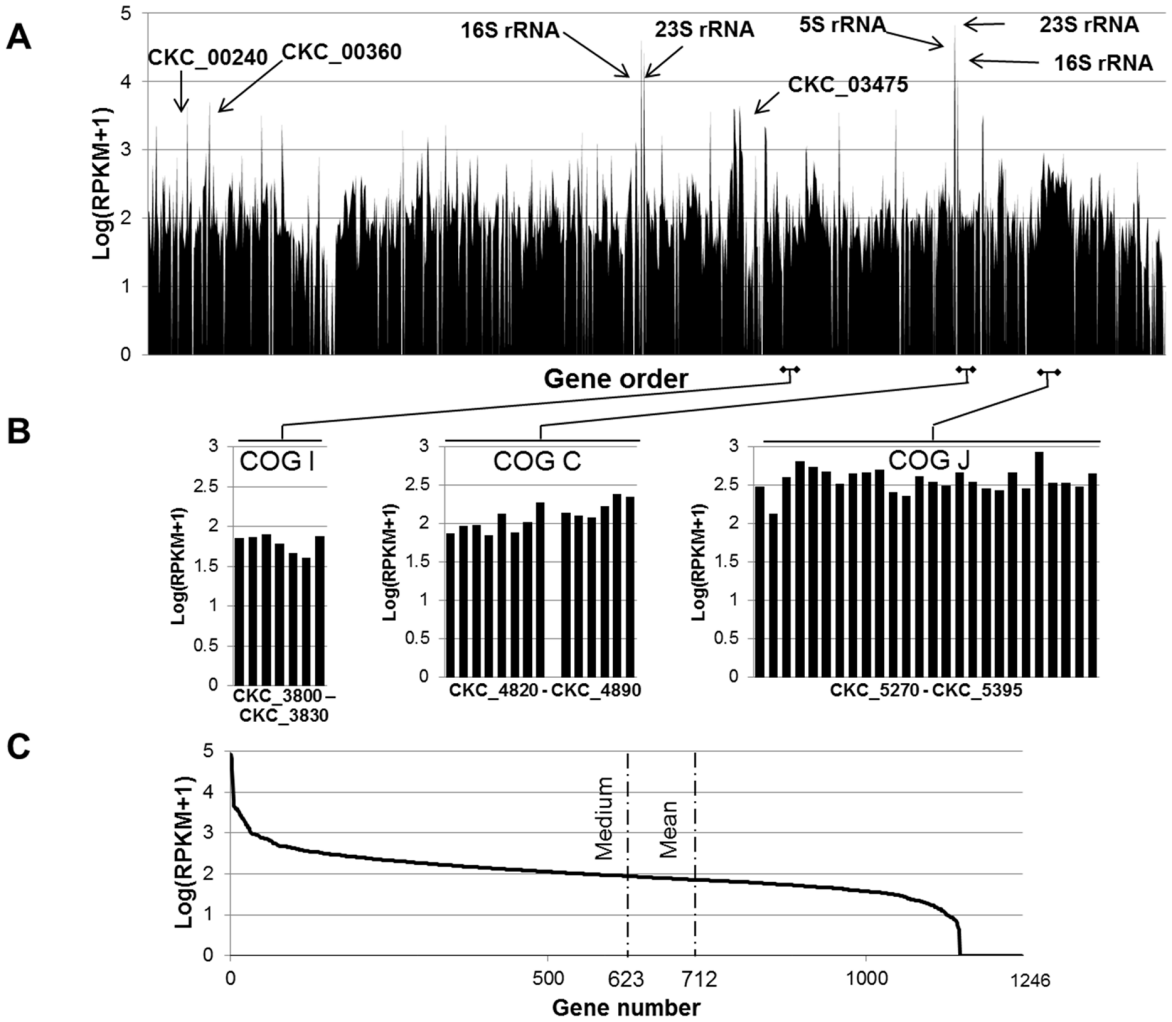
Quantification of Lso gene expression in its vector. **A**. Gene expression across Lso genome. The x-axis shows the gene order and the y-axis shows log(RPKM+1) value for each gene. **B**. Three genome regions with clusters of genes involved in a similiar COG function and with similar gene expression level. **C**. Distribution of gene expression abundance sorted from high to low expression value, x-axis shows the gene number and y-axis shows log(RPKM+1) value. Dashed lines represent mean and medium values.

Expression levels were quantified by calculating RPKM (reads per kilobase CDS length per million reads analyzed) values for each gene (Table S1). Gene expression across the genome can be visualized as a gene expression map using log(RPKM+1) values (Figure 2A). The mean and medium log(RPKM+1) values were 1.87 and 1.95, respectively, indicating a slight skew toward lowly expressed genes (Figure 2C): 712 genes were expressed at higher level than the mean expression value 1.87, while 534 genes were expressed at lower level. Of the 1246 Lso predicted genes, we mapped reads to 1148 of them (92%). Gene clusters involved in a similar function showed similar levels of gene expression (Figure 2B). For example, genes within the region between CKC_05270 and CKC_05395 encoding Ribosomal proteins were expressed at higher level [log(RPKM+1)>2.14] compared to genes between CKC_3800 and CKC_3830 [log(RPKM+1)<1.9] involved in lipid metabolism.

The top most expressed genes were 5S rRNA, 16S rRNA, and 23S rRNA. Among the top 10 most expressed coding sequences (CDS) were cold and heat shock proteins, outer membrane protein, ferroxidase, GroES and hypothetical proteins (Table 6). Our results are in accordance to other studies on bacterial endosymbionts, for instance *Wolbachia* transcriptome in nematodes [39] and *B. aphidicola* in aphids also revealed high expression of chaperones associated with heat-shock responses. In particular, it has been established that GroEL proteins are abundantly produced by many bacterial endosymbionts [39], [40], [41], [42]; and GroEL and its co-chaperone GroES have been proposed to alleviate the deleterious mutations that are irreversibly fixated by genetic drift in endosymbionts [43]. Since Lso can be acquired by insects vertically and horizontally (through feeding from an infected host) we could hypothesize that deleterious mutations might not be fixated at the same rate than in exclusively vertically-transmitted bacteria. However, high expression of chaperones was also measured in some free living bacteria [44], [45].

**Table 6:** Top 10 most expressed Lso CDS.

CDS	Log(RPKM+1)
Small heat shock protein CKC_00360	3.80
Hypothetical protein CKC_03475	3.66
Outer membrane protein CKC_00240	3.63
Hypothetical protein CKC_03440	3.60
Hypothetical protein CKC_03455	3.59
Hypothetical protein CKC_04425	3.59
Cold shock protein CKC_03300	3.56
Hypothetical protein CKC_04080	3.55
GroES CKC_04900	3.51
Ferroxidase CKC_00675	3.50

No expression was detected for 98 genes [log(RPKM+1) = 0]. Among those genes were the 45 tRNA genes. An RPKM value of 0 was expected for those genes since tRNAs are short (∼75 nucleotides long on average) and the parameters for RPKM mapping used in this study were 0.9 minimum length of the read mapped per gene; since reads were 100 nucleotides long it is impossible that 90% of the read could match a predicted tRNA gene. Therefore, RPKM values of bacterial endosymbiont genes that might have undergone reduction of gene length could be underestimated. When mapping reads to Lso genome we found reads mapping to 44 of the tRNA genes.

Coding sequences for which no transcripts were detected included 44 genes encoding hypothetical proteins (13 of which were less that 100 bp long), 5 phage-related genes (2 genes encoding P4 family phage/plasmid primase, a gene encoding a phage-related integrase/recombinase, a prophage antirepressor, and a putative DNA polymerase from bacteriophage). The other 4 CDS with no detected transcripts were CKC_0190 encoding a VRR-NUC domain-containing protein, CKC_0195 encoding SNF2 related protein, CKC_01385 encoding DNA polymerase III subunit delta, and CKC_3590 encoding uracil-DNA glycosylase.

CKC_3590 encodes a 42 AA long uracil-DNA glycosylase. Two other genes encoding uracil-DNA glycosylases were annotated in Lso genome: the neighboring gene CKC_3585 encoding a 148 AA long protein and CKC_0780 encoding a 260 AA long protein. Both, CKC_3585 and CKC_0780, were expressed in our analysis based on the log(RPKM+1) values, 1.66 and 1.86, respectively. Analysis of read mapping showed 2 reads mapping CKC_3590, but only 68 nucleotides overlapped the predicted CDS, which is lower than the cut-off parameters, as previously discussed. Interestingly, both reads show a one nucleotide indel compared with the genomic sequence. This indel induces a frame shift, as a result CKC_3590 and CKC_3585 are in the same frame and they encode a single 225 AA long protein. Resequence of the Lso genomic DNA is needed to confirm the existence of this indel which could help to improve Lso genome annotation.

Similarly, 2 genes encoding DNA polymerase III subunit delta were found: CKC_01385 and its neighbor CKC_01380, encoding a 113 AA and a 86 AA protein long, respectively. While no gene expression was detected for CKC_01385, log(RPKM+1) value for CKC_01380 was 1.22. Interestingly, it appears that CKC_01380 and CKC_01385 encode two portions of a unique DNA polymerase III subunit delta protein: the analysis of Lso genome sequence revealed a frameshift in the gene encoding DNA polymerase III subunit delta resulting in the two predicted genes. Our transcriptome analysis showed reads mapping to CKC_01380 as well as the region in between both CDS and part of CKC_01385. However, as previously only 77 nucleotides of the read overlapped with the predicted gene, which is below the cut-off parameter when calculating RPKM values.

Two other genes for which no gene expression was detected were the neighbors CKC_01090 and CKC_01095 encoding a VRR-NUC domain-containing protein and a SNF2 related protein, respectively. Remarkably, CKC_05960 and CKC_05965 also encoded a VRR-NUC domain-containing protein and a SNF2 related protein, respectively, and their expression levels were 1.44 and 1.61, respectively. Each of these regions is 1672 nucleotides long, and they differ from each other by 26 nucleotides. It is interesting to note that both regions might be expressed in different conditions.

### Functional analysis of Lso gene expression in its vector

**Figure 3:**
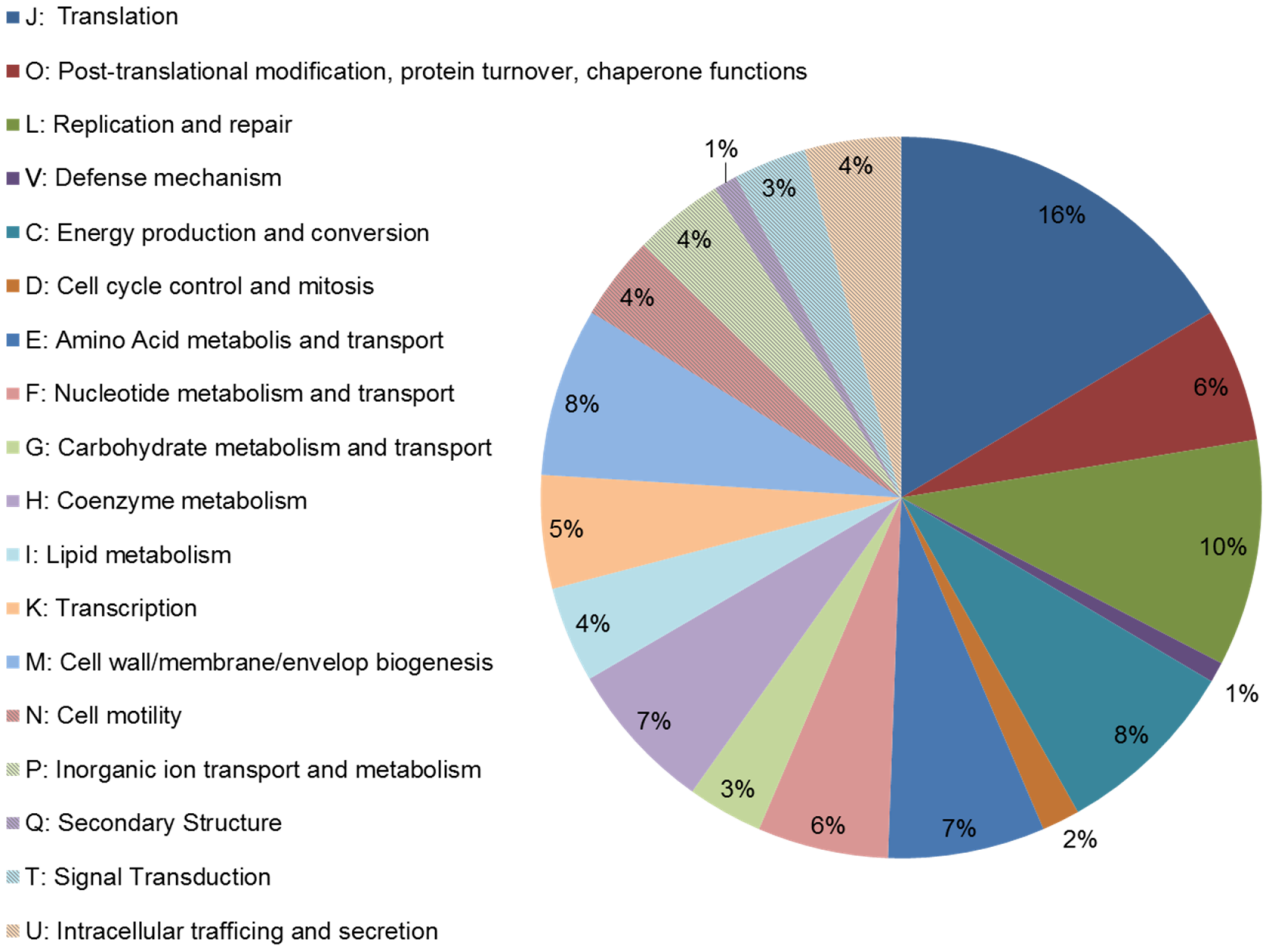
Distribution of Clusters of Orthologous Groups (COG) in Lso transcriptome when associated with its insect vector (R and S are not included).

Classification of genes into Cluster of Orthologous Groups (COG) indicated that all categories had a mean log(RPKM+1) value between 1.74 and 2.28. Genes involved in Translation (COG: J) were among the most expressed genes based on number of expressed genes in the category (126 genes, 16% of the expressed genes, Figure 3) or the mean log(RPKM+1) value, 2.15. The category with the highest mean log(RPKM+1) value (2.28) was Post-translational modification, protein turnover, chaperone functions (COG: O) which contained 46 expressed genes (6% of the expressed genes). Whereas the category Replication and repair (COG: L) represented the second largest expressed category with 78 expressed genes (10%) but had the lowest mean log(RPKM+1) value, 1.74. Interestingly, 7 genes belonging to Defense mechanisms (COG: V) were identified, all of them were expressed with a mean log(RPKM+1) value of 1.77. Nucleoside and nucleotide metabolism are proposed to support nutritional provisioning of *Wolbachia* associated with nematodes, and the bacterial transcriptome analysis confirmed high expression level of genes in that category [39]. Therefore, our results point to the importance of translation (COG J), and post-translational modification, protein turnover and chaperone functions (COG O) in Lso associated with psyllids. It would be interesting to compare the expression level of Lso genes when the bacterium is associated with the host plant.

**Figure 4:**
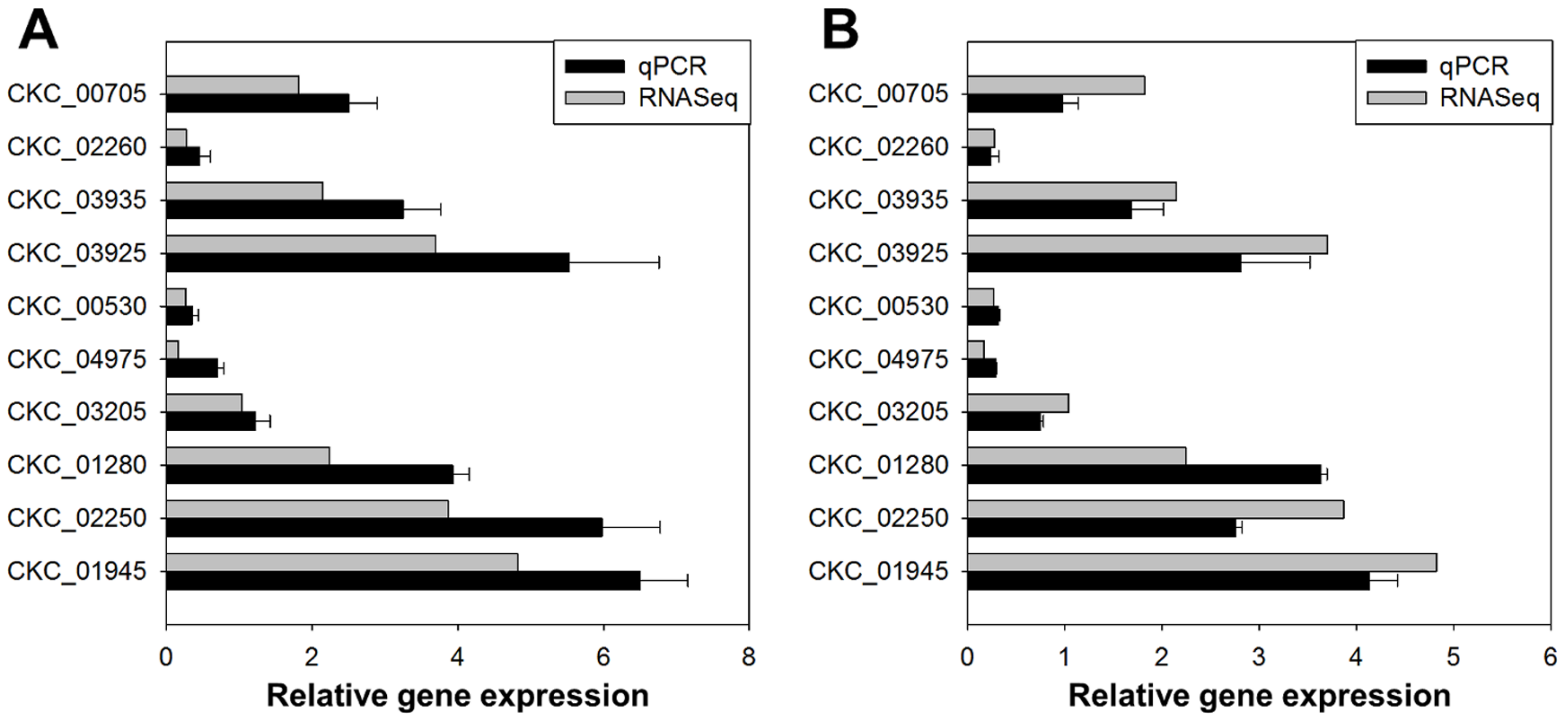
Validation of RNA-seq results by RT-qPCR. **A**: Same rRNA depleted RNA sample used for Illumina sequencing was used for RT-qPCR. **B**: Three different biological replicates were used for RT-qPCR. Relative gene expression levels of 10 selected Lso genes normalized to the expression value of the Lso *recA* gene. Black bars: for each gene transcript expression value was determined by RT-qPCR; data points represent means ± SD of three replicates. Grey bars: for each gene transcript expression value was determined using the RPKM values.

To validate if calculated RPKM values were a good representation of gene expression, we performed RT-qPCRs for 10 Lso genes (Table 2) chosen to represent a range of gene expression from high to low using the same RNA-depleted RNA sample submitted for Illumina sequence. We further validated these results by testing gene expression in three different biological replicates. We performed a linear correlation analysis of the relative expression levels between each RT-qPCR data for each experiment and the RPKM values (Figure 4). Our results indicated a strong Pearson correlation coefficient r = 0.9813 (p<0.005) which shows that in this case, combining reads from the PolyA and the depleted RNA samples did not introduce a bias. However, this might not be true for other genomes with a higher or a lower C+G content. Similarly, a strong Pearson correlation coefficient r = 0.8096 (p<0.005) was found between the bioinformatics results and the three different biological replicates.

### Transcriptomic data to improve genome annotation

**Figure 5:**
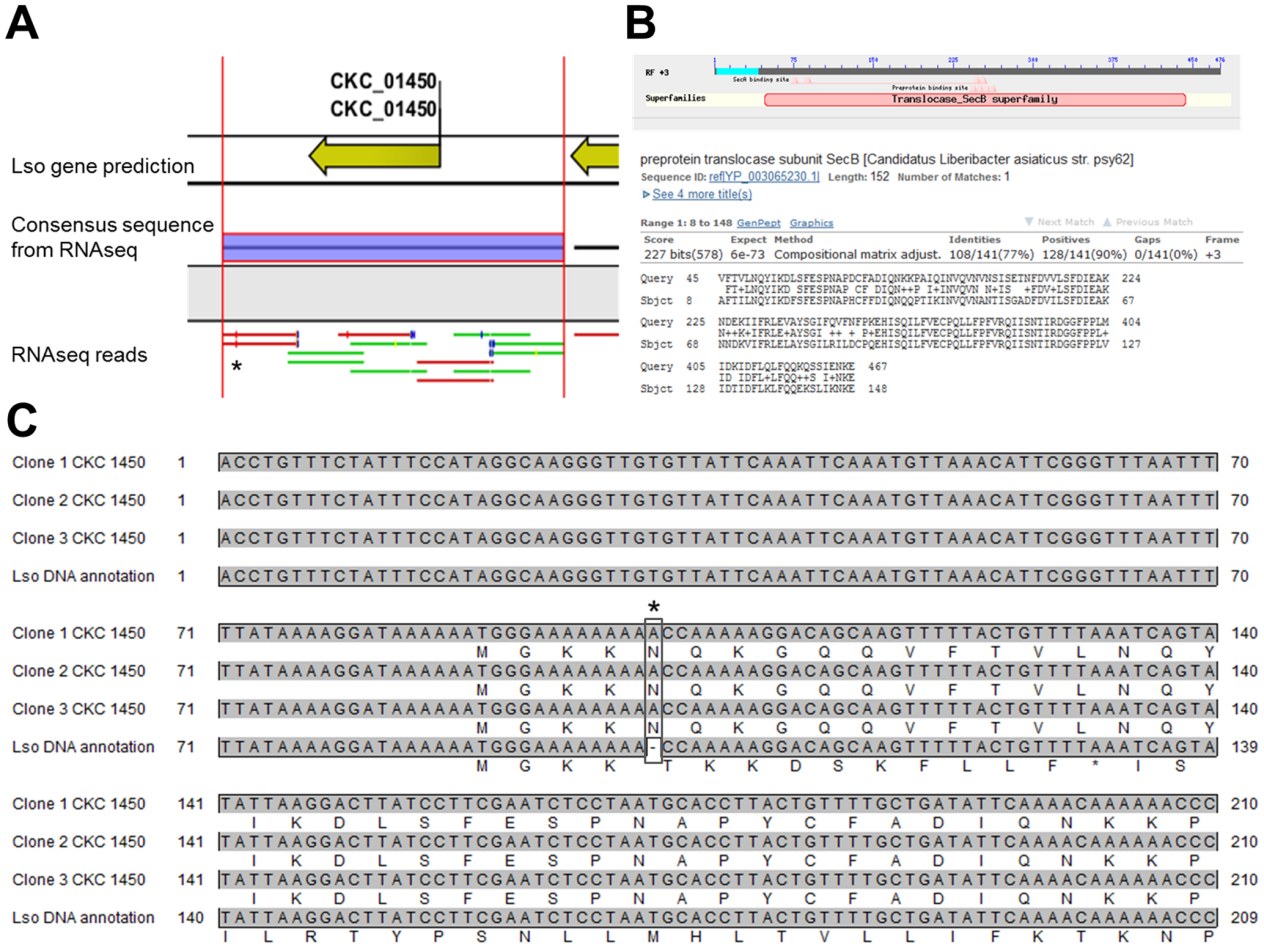
Prediction of *secB* gene in Lso genome. **A**. Screenshot of CLC genomic workbench 4.8 platform: Yellow arrows represent Lso gene predictions, purple bar represents the consensus sequence predicted from RNAseq reads (green and red bars). * shows extra base in RNAseq reads absent in Lso genome sequence. **B**. BLASTX search of the consensus sequence identified a match with “*Candidatus* Liberibacter asiaticus” SecB protein. **C**. Alignment of Lso genome and 3 cloned sequences for the N′ half of the *secB* gene and their *in silico* translated sequences. Frameshift region is shown by *.

Transcriptomic data can be used to identify putative CDS not predicted by *in silico* analyses. For instance, a putative gene, not identified by the *in silico* annotation of Lso genome, was identified by read mapping (Figure 5). Several reads mapped upstream and downstream CKC_01450. Translation of the obtained consensus sequence yielded 2 ORFs, a 60 AA long protein in the reverse strand (encoded by CKC_01450) and an unpredicted 151 AA long protein with similarity to preprotein translocase subunit SecB from Las (E value e-73) in the forward strand. *SecB* gene had not been found previously in the Lso genome [12]. A missing Adenine, 11 nucleotides after the initial ATG in the genome created a frameshift, probably explaining why this gene was not predicted. Interestingly, the transcriptomic reads mapping this region contained this extra nucleotide (* in Figure 5). To investigate if our transcriptomic result arose from a sequencing error, the genomic region surrounding the putative *secB*-like genome was amplified, cloned and sequenced (accession number: KJ596483). Our results confirmed that Lso harbored by the psyllids in this study might encode a putative SecB protein. Expression of the putative *secB* was confirmed by RT-PCR (Figure S2).

**Figure 6:**
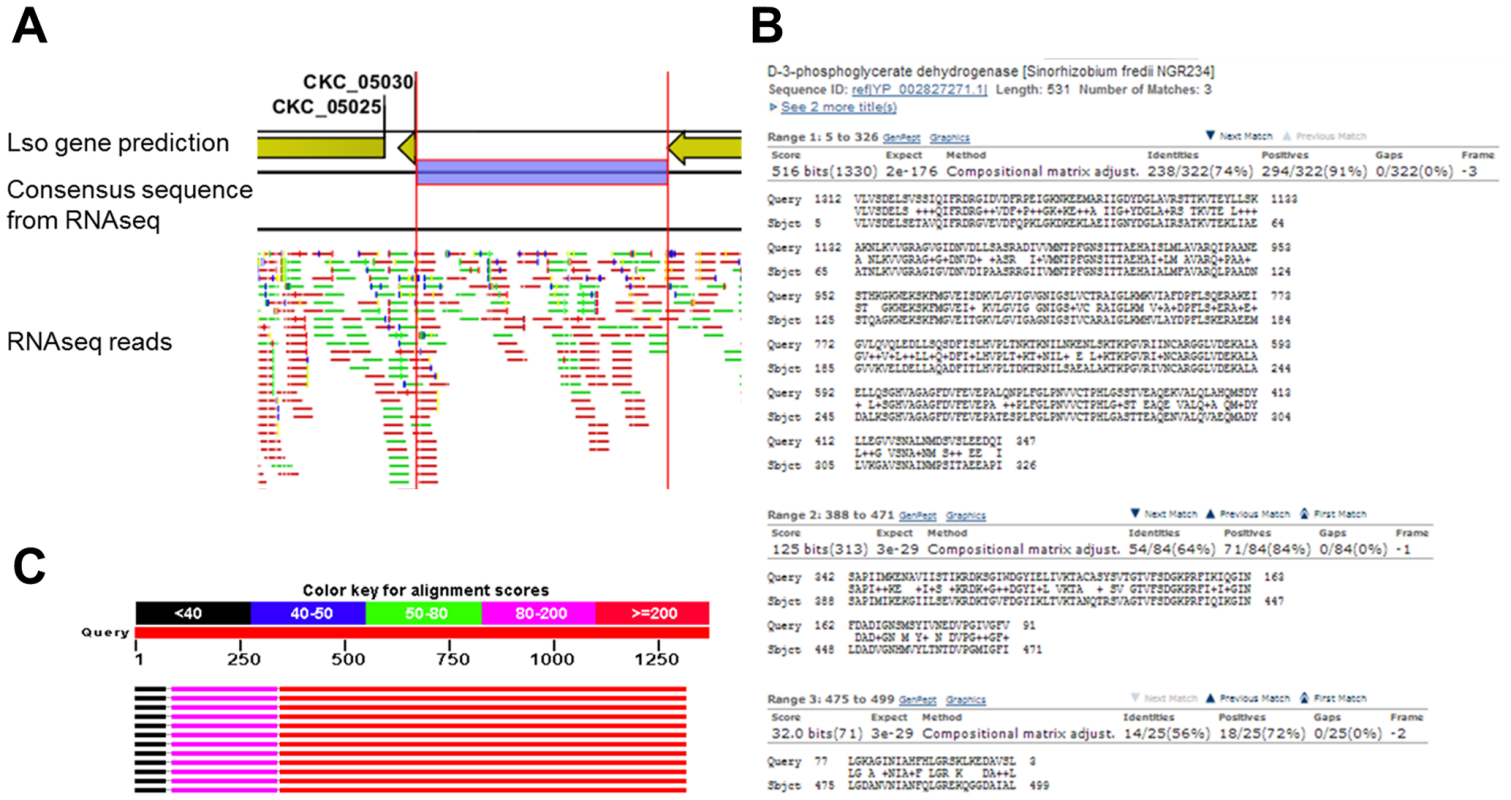
Prediction of *serA* gene in Lso genome. **A**. Screenshot of CLC genomic workbench 4.8 platform: Yellow arrows represent Lso gene predictions, purple bar represents the consensus sequence predicted from RNAseq reads (green and red bars). **B and C**. BLASTX search of the consensus sequence identified a match with *Sinorhizobium fredii* PGDH protein.

Similarly, our study might have identified a putative *serA*, a gene encoding D-3-phosphoglycerate dehydrogenase, an enzyme that catalyzes the first step in serine biosynthesis (Figure 6). Several reads mapped between genes CKC_05030 and CKC_05045 (CKC_05045 is predicted to be a phosphoserine aminotransferase). Similarity searches using Blastx revealed that this region showed similarities to D-3-phosphoglycerate dehydrogenase (PGDH) (top hit: *Sinorhizobium fredii* NGR234 Evalue = 6e-176, 521AA). However, consistent with the Lso genome sequence our consensus sequence showed 2 frameshifts that interrupt the gene which would encode a 327 AA protein. We cloned and sequenced the putative gene and verified that the first frameshift occurs, resulting in a shorter protein (accession KJ609520). Expression of this putative CDS was confirmed by RT-PCR analyses (Figure S2). *Escherichia coli* encodes a 410 AA long PGDH protein [46] composed by three domains: nucleotide-binding domain (residues 108–292), substrate binding-domain (residues 1–102, 304–318) and regulatory domain (residues 336–410) [47]. L-serine inhibits the activity of the enzyme [48] allosterically by binding the regulatory domain. Truncation analyses have shown that the removal of the regulatory domain did not affect the enzymatic activity but eliminated the serine inhibition [47]. It cannot be excluded that Lso encodes an active PGDH lacking the regulatory domain, in which case it would be interesting to understand why that regulation is not necessary.

**Figure 7:**
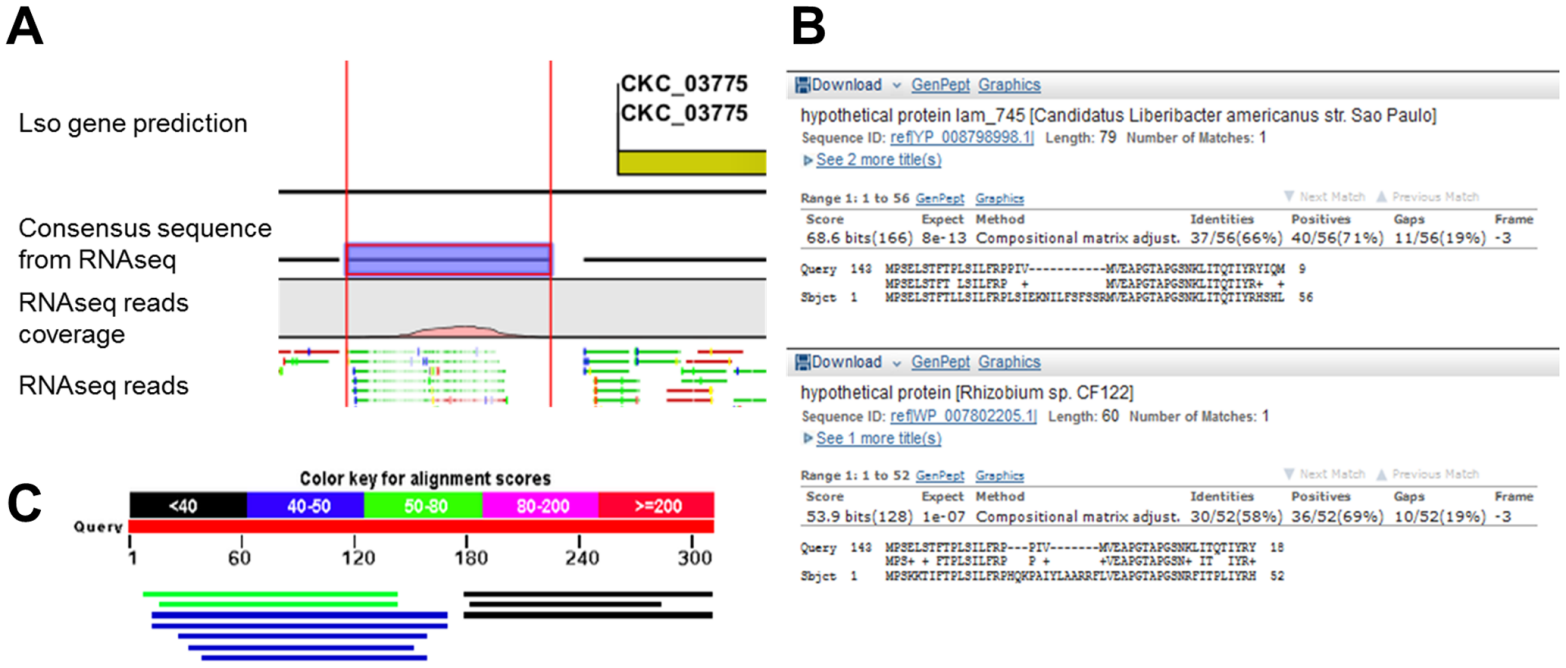
Prediction of a gene encoding a Hypothetical protein in Lso genome. **A**. Screenshot of CLC genomic workbench 4.8 platform: Yellow arrows represent Lso gene predictions, purple bar represents the consensus sequence predicted from RNAseq reads (green and red bars). **B and C**. BLASTX search of the consensus sequence identified a match with “*Candidatus* Liberibacter americanus” and with *Rhizobium* sp. Hypothetical proteins.

Finally, a different example of putative unpredicted gene is presented in Figure 7. In this case 5,390 reads mapped between CKC_03770 and CKC_03775. *In silico* analysis of the region revealed the existence of least 1 ORF yielding a 59 AA long protein showing similarities to Hypothetical proteins predicted in different Rhizobiaceae. DNA sequencing and RT-PCR analyses confirmed the sequence of the putative gene and its expression (Figure S2).

## Conclusions

The current study represents the first transcritpomic analysis of Lso in its insect vector, *B. cockerelli*. The key features identified in this analysis are the high percentage of CDS expressed in the vector, the homogeneity of expression level across COG categories but the high expression level of putative genes involved in translation and in post-translational modification, protein turnover, chaperone functions. Confirmatory RT-qPCR of genes expressed at different levels based on RPKM predictions validated the relative expression level of 10 target genes. In addition, mapping of reads to Lso genome allowed to identify new CDS not previously predicted *in silico*, among which *secB* and *serA* as well as several putative short genes (less that 180 base pairs). This study also revealed the limitations resulting from the length of the Illumina reads. In this case, long Illumina reads were chosen because the insect harbors several bacteria with no available genome and long reads allowed to better assign the organism of origin. On the other hand, long reads resulted in an underestimation of the expression of short genes. As more genomes of microbes associated with the host are made available, this issue should be less important. Finally, the advent of RNAseq will advance the study bacterial gene expression within hosts and unravel the molecular interactions between microbes and hosts (primary or vector hosts).

## Supporting Information

Figure S1 **PCR amplification products to determine presence of bacteria associated with the psyllids used in this study.** A: Detection of *C. ruddii*. B: Detection of *Wolbachia*. C: Identification of Lso haplotypes carried by psyllids from the donor colony. Lanes 1 DNA ladder; lanes 2 to 9: single psyllid from donor colony; lanes 10 (A and B): positive control; lanes 11 (A and B): negative control; lane 10 (C): positive control Haplotype A (237 bp); lane 11: positive control haplotype B (169 bp); lane 12 (C): negative control.
(TIF)

Figure S2 **RT-PCR amplification products to determine expression of putative new genes using three different biological samples.** Lane 1: DNA ladder; lanes 2-4: *hypothetical protein*, lanes 6–8: *secB*; lanes 10–12: *serA*; lanes 5, 9 and 13: negative controls.
(TIF)

Table S1 **Complete list of expression values for Lso annotated genes.**
(XLSX)
