# Supplementary figures and images for: Transcriptome Analysis of “Candidatus Liberibacter solanacearum” in Its Psyllid Vector, Bactericera cockerelli
Source: PLoS One. 2014 Jul 3;9(7):e100955. doi: 10.1371/journal.pone.0100955 (PMC4081026; doi:10.1371/journal.pone.0100955)

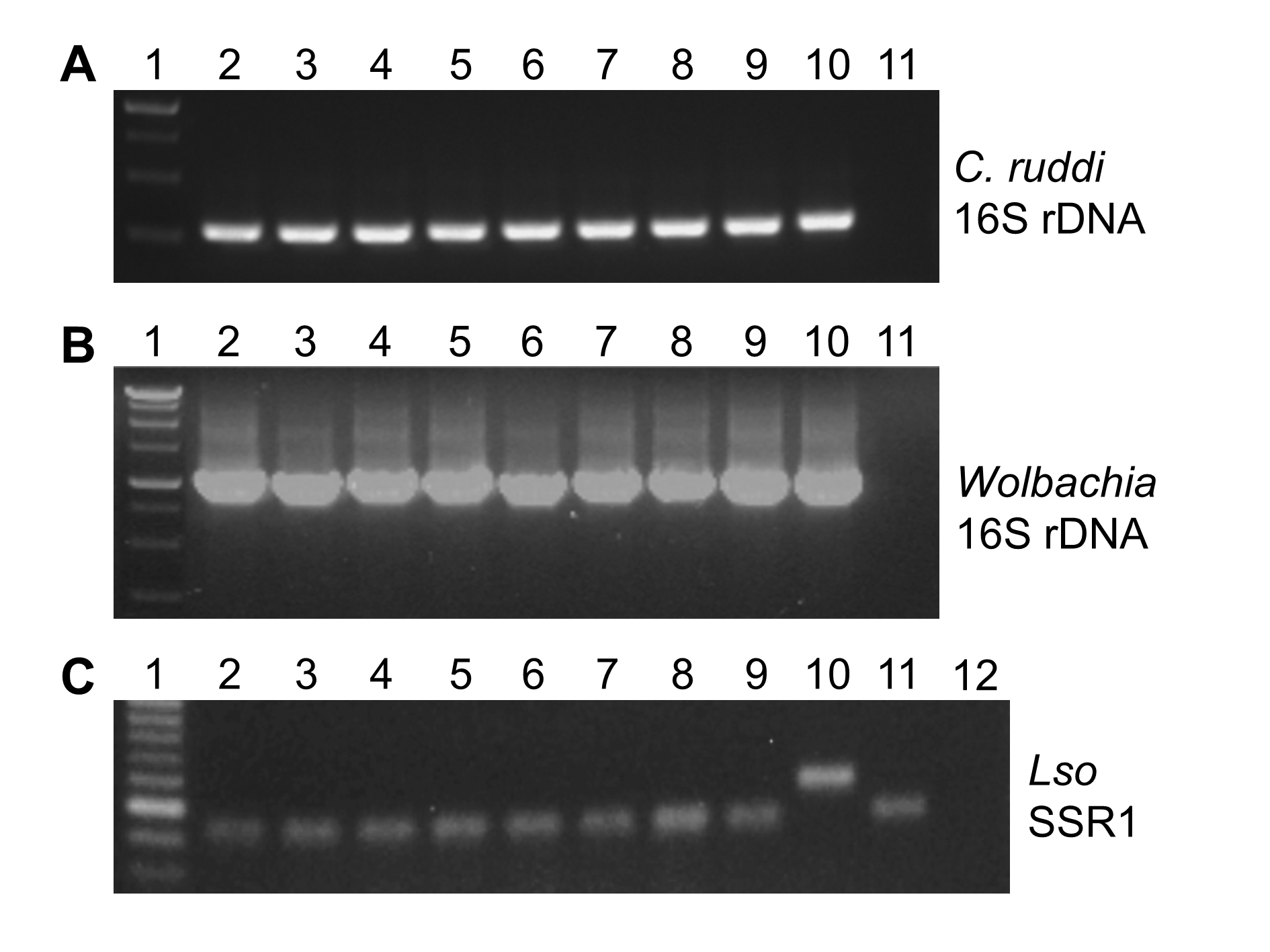

Supplement: Figure S1 — PCR amplification products to determine presence of bacteria associated with the psyllids used in this study. A: Detection of C. ruddii. B: Detection of Wolbachia. C: Identification of Lso haplotypes carried by psyllids from the donor colony. Lanes 1 DNA ladder; lanes 2 to 9: single psyllid from donor colony; lanes 10 (A and B): positive control; lanes 11 (A and B): negative control; lane 10 (C): positive control Haplotype A (237 bp); lane 11: positive control haplotype B (169 bp); lane 12 (C): negative control. (TIF) [file pone.0100955.s001.tif]

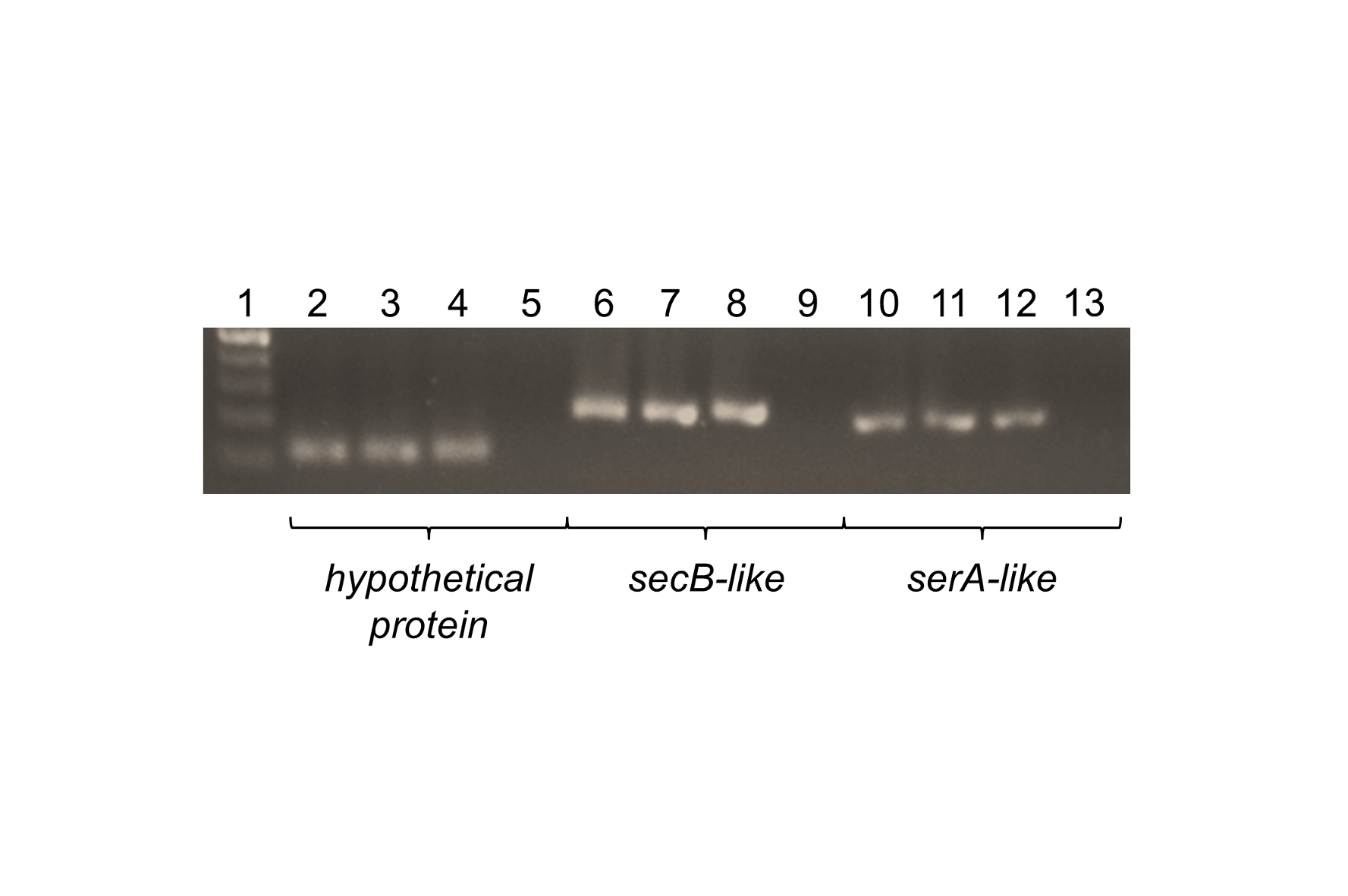

Supplement: Figure S2 — RT-PCR amplification products to determine expression of putative new genes using three different biological samples. Lane 1: DNA ladder; lanes 2-4: hypothetical protein, lanes 6–8: secB; lanes 10–12: serA; lanes 5, 9 and 13: negative controls. (TIF) [file pone.0100955.s002.tif]
